# Is the Oxidative DNA Damage Level of Human Lymphocyte Correlated with the Antioxidant Capacity of Serum or the Base Excision Repair Activity of Lymphocyte?

**DOI:** 10.1155/2013/237583

**Published:** 2013-11-14

**Authors:** Yi-Chih Tsai, Pei-Yi Li, Chung-Chu Chen, Yin-Chang Liu

**Affiliations:** ^1^Institute of Molecular Medicine, National Tsing-Hua University, Hsinchu 30013, Taiwan; ^2^Department of Pathology and Laboratory Medicine, Hsinchu Mackay Memorial Hospital, Hsinchu 30013, Taiwan; ^3^Department of GI and Hepatology, Hsinchu Mackay Memorial Hospital, Hsinchu 30013, Taiwan

## Abstract

A random screening of human blood samples from 24 individuals of nonsmoker was conducted to examine the correlation between the oxidative DNA damage level of lymphocytes and the antioxidant capacity of serum or the base excision repair (BER) activity of lymphocytes. The oxidative DNA damage level was measured with comet assay containing Fpg/Endo III cleavage, and the BER activity was estimated with a modified comet assay including nuclear extract of lymphocytes for enzymatic cleavage. Antioxidant capacity was determined with trolox equivalent antioxidant capacity assay. We found that though the endogenous DNA oxidation levels varied among the individuals, each individual level appeared to be steady for at least 1 month. Our results indicate that the oxidative DNA damage level is insignificantly or weakly correlated with antioxidant capacity or BER activity, respectively. However, lymphocytes from carriers of *Helicobacter pylori* (HP) or *Hepatitis B virus* (HBV) tend to give higher levels of oxidative DNA damage (*P* < 0.05). Though sera of this group of individuals show no particular tendency with reduced antioxidant capacity, the respective BER activities of lymphocytes are lower in average (*P* < 0.05). Thus, reduction of repair activity may be associated with the genotoxic effect of HP or HBV infection.

## 1. Introduction

The endogenous level of DNA damage due to oxidative stress in human peripheral blood lymphocytes (PBL) has been extensively used as biomarkers in studying the genotoxic effects associated with diseases, microbial infection, ageing, or the exogenous agents [1–7]. However, the DNA damage levels presented in the previous studies were often collected at a single point time; it is unclear whether the damage level of concern is steady for a certain period of time, for example, a week or a month or longer. In this report, we showed that the endogenous level of DNA oxidation in lymphocytes from each individual was constant at least for 1 month (see the following). We measured the level of DNA oxidation in lymphocytes with a modified comet assay, which includes a step of enzymatic cleavage by bacterial Fpg/Endo III, recognizing oxidized purines and pyrimidines, respectively [8, 9]. We considered that such damage levels of PBL may be modulated by the antioxidant capacity of serum or the repair activity of lymphocytes. Like the measurement of DNA damage in PBL, serum or plasma antioxidant capacity has long been used as biomarker for various studies [10]. In contrast, the repair activity of PBL as biomarker just began to receive attention [11]. The repair activity can be assessed with plasmids damaged with specific agents or the synthetic oligonucleotides specific for particular lesions [12, 13]. Repair of the DNA lesions (including excision and gap-filling activities) is indicated by the incorporation of P^32^-labled nucleotide. On the other hand, the activity to excise oxidative DNA lesions can be evaluated with a modified version of comet assay, in which the cell or nuclear extract is used to replace the Endo III/Fpg. Cells (not for extracts) treated with a constant amount of H_2_O_2_ or other oxidative agents are embedded in gels and used as substrate for the purpose [14, 15]. 

To test if the steady state levels of oxidative DNA damage of PBL are modulated by the antioxidant capacity of serum or the repair activity of PBL, 24 peripheral blood samples obtained from the individuals for routine health check-up were examined. Our results indicate that the level of oxidative DNA damage in PBL is not correlated with the serum antioxidant capacity but is weakly correlated with the BER activity in lymphocyte NE. Also, we found that PBL from carriers of *Helicobacter pylori* or *Hepatitis B virus* had higher levels of oxidative DNA damage, to which the BER activity but not the serum antioxidant capacity may attribute. 

## 2. Materials and Methods

### 2.1. Patients and Samples

**Figure 1:**
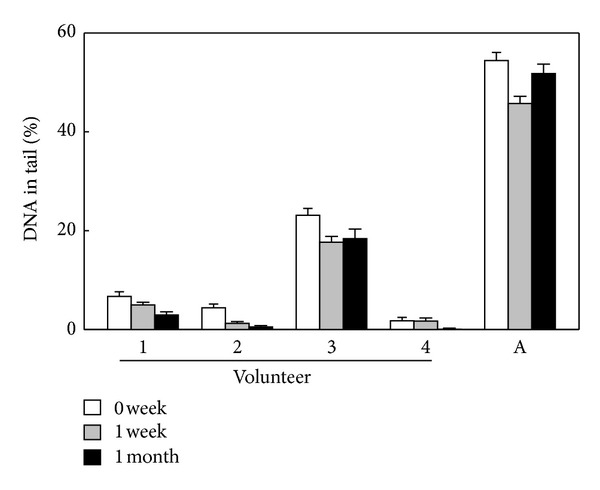
Oxidative DNA damage level of lymphocyte is steady. Lymphocyte samples of four volunteers collected at the indicated period of time were analyzed for the oxidative DNA damage with comet-Fpg/Endo III assay. 1–4: individual labels of the four volunteer; A: human AGS cells treated with 5 mM amoxicillin for 1 h, a positive control for enzyme (Fpg/Endo III) activity.

The 24 blood samples were randomly collected from individuals (males and females) who were 20–40 years old, nonsmoking, nondrinking, and lived/worked in similar environment. The four volunteers (males and females) for the experiment described in Figure 1 were 25–35 years old, nonsmoking, nondrinking, apparently healthy, and lived/worked in similar environment. The study was approved by Institutional Review Board of Taiwan (13MMH IS 189).

### 2.2. Isolation of the Lymphocytes

Lymphocyte were isolated from blood samples with the modified procedures of those described previously [16]. The blood samples were centrifuged at 3000 rpm (1400 ×g) for 15 min at 22°C for separation of serum from blood cells, and the serum on the top of the centrifuge tube was collected for antioxidant capacity measurement. The cells at the bottom of the centrifuge tube were resuspended in PBS at 1 : 1 ratio and the suspension was layered at 4 : 3 ratio to the ficoll-paque PLUS (GE Healthcare 17-1440-02) in another centrifuge tube. The whole assembly of blood cells was centrifuged at the same experimental condition described previously and the lymphocytes were collected from the layer between plasma and ficoll-paque layers. The cells were washed twice with PBS before analyses. One part of the cells was used for oxidative DNA damage experiment and the other part of the cells was used for repair activity experiment. 

### 2.3. Detection of Oxidative DNA Damage with the Comet-EndoIII/Fpg Assay

The method described previously [17] was followed. In brief, conventional alkaline comet assay procedures were performed to obtain nucleoids in agarose gels on slides. Each slide was incubated with 2 units of both EndoIII and Fpg (Trevigen, Inc., Gaithersburg, MD, USA) in buffer of 10 mM Tris-HCl pH 7.4 for 1 h at 37°C in a sealed, humid chamber. EndoIII and Fpg are bacterial glycosylases that specifically recognize oxidized pyrimidines and purines, respectively. After enzyme digestion, DNA in nucleoids was alkaline denatured and then separated with electrophoresis. Nucleoids after staining with propidium iodide were examined with a fluorescence microscope; images of at least 50 nucleoids per slide were recorded with a closed-circuit display CCD camera (Zeiss/Axioskop 2 Mot plus). The migration of DNA from the nucleus of each cell was measured with a computer program (http://tritekcorp.com/) and is expressed as % DNA in the tail.

### 2.4. Trolox Equivalent Antioxidant Capacity (TEAC) Assay

The standard TEAC assay described in [18] and in [19] has been used with minor modifications for determination of the TEAC value. This assay assesses the total radical scavenging capacity based on the ability of a compound to scavenge the stable ABTS (Sigma Aldrich, NO. A9941-5TAB) radical (ABTS^•^) in 6 min. The blue-green ABTS^•^ was produced through the reaction between 7 mM ABTS and 2.45 mM potassium persulfate in water. This solution was stored in the dark for 12–16 h at 4°C before use. The extinction coefficient of ABTS^•^ at 734 nm is 1.5 × 10^4^ mol^−1^ l cm^−1^. The concentrated ABTS^•^ solution was diluted with phosphate buffered saline (PBS), pH 7.4 to a final absorbance of 0.8 ± 0.02 at 734 nm at 37°C (i.e., an ABTS^•^ concentration of approximately 47 *μ*M). Stock solutions of trolox (Sigma Aldrich, NO. 23881-3) were prepared in ethanol and serve as the standard curve. For the samples, 100 *μ*L serum were added to 900 *μ*L ABTS^•^ solution and the absorbance at 734 nm was measured in time. This was compared to a blank where 100 *μ*L of the PBS was added to 900 *μ*L of the ABTS^•^ solution. The initial reduction of absorbance was determined 3 min after addition of the antioxidant with constant mixing time (about 10 sec). The TEAC of serum was calculated by relating this decrease in absorbance to that of variation trolox concentrations served as a standard curve and normalized with protein amount determined with BCA kit (Pierce Chemical Co., Chester, UK).

### 2.5. Preparation of Nuclear Extract (NE) from Lymphocytes and Comet-NE Assay to Determine the Base Excision Activity in NE of Lymphocytes from 24 Blood Samples

Preparation of nuclear extract for comet-NE assay (see below) is done as described in our previous study [17]. In brief, the isolated lymphocytes were first treated with 2.5 mM hydroxyurea and 25 *μ*M cytosine-*β*-d-arabinofuranoside for 16 h. Hydroxyurea, an inhibitor of ribonucleotide reductase, and cytosine-*β*-d-arabinofuranoside, an inhibitor of DNA polymerase, are used here to prevent DNA synthesis in comet-NE assay. The cells were washed with hypotonic buffer (20 mM Hepes, pH 7.5, 5 mM KCl, 0.5 mM MgCl_2_, 0.5 mM dithiothreitol, and 0.2 M sucrose) and were allowed to swell in the hypotonic buffer without sucrose for 10 min on ice. The swollen cells were then ruptured with 10 strokes of a Dounce homogenizer and homogenates were forced through a 22 G needle 10 times. Each mixture was centrifuged at 2000 ×g for 5 min and nuclear pellets were resuspended in buffer (20 mM Hepes, pH 7.5, 5 mM KCl, 0.5 mM MgCl_2_, 0.5 mM dithiothreitol, and 10% sucrose) and stored at −70°C. The nuclei were thawed on ice and allowed to swell in 100 mM NaCl on ice for 1 h. The ruptured nuclei were centrifuged at 15,000 ×g for 20 min at 4°C, and the supernatants were filtered through a YM-10 Microcon filter (Millipore, Bedford, MA, USA). Protein concentrations were determined with a BCA Protein Assay Kit; bovine serum albumin was used as a standard.

The procedures of Comet-NE assay are similar to comet-EndoIII/Fpg assay unless in the enzyme incubation NE of lymphocytes but no Endo III/Fpg was performed. Constant amount of NE (about 0.6 *μ*g) in NE buffer contained 50 mM Hepes-KOH (pH 7.9), 70 mM KCl, 5 mM MgCl_2_, 0.4 mM EDTA, 2 mM ATP, and 40 mM phosphocreatine, and 2.5 mM creatine phosphokinase was added on each slide. Also, to determine base excision activity, the amoxicillin-treated (5 mM for 1 h) human AGS cells were used as the cell substrate in the comet-NE assay. Previously, we have reported that amoxicillin caused oxidative DNA damage [8, 20]. 

### 2.6. Cell Cultures

The AGS human gastric adenocarcinoma cell used in this study were originally obtained from the American Type Culture Collection (Manassas, VA, USA). AGS cells were grown in 1X RPMI medium (Sigma Aldrich, NO. R6594). All cell culture media were supplemented with 10% fetal bovine serum (FBS; Sigma Aldrich, NO. 19003C), and 0.03% glutamine and cells were grown at 37°C in a water-saturated atmosphere containing 5% CO_2_.

### 2.7. Immunological Test

Infection of *Helicobacter pylori* (HP) or carriers of *hepatitis B virus* (HBV) were identified with DPC IMMULITE 2000 (Global Siemens, Germany) tested for presence of IgG against *H. pylori* or surface antigen of HBV.

### 2.8. Data Analysis

All experiments were performed independently at least three times. Data are expressed as means ± SE. Student's *t*-test was used for statistical analyses. A *P* value of <0.01 is considered to be statistically significant.

## 3. Results

### 3.1. The Oxidative Damage Level of Lymphocyte Is Stable

Although the oxidative DNA damage levels of human lymphocytes have been used as biomarker for investigating the intervention of antioxidants, often the measurement was done as the single time point. Whether the oxidative DNA damage level of lymphocyte from an individual is steady over a period of time, for example, months, remains unclear. Thus, we conducted an experiment involving four apparently healthy volunteers, and use the comet-*EndoIII/Fpg* assay to monitor the DNA damage level of lymphocyte for a month. The results indicate that the oxidative damage levels varied among the individuals; however, levels of each individual were relatively steady over a month unless a slightly decreasing trend of the damage levels of individuals was noted over the period of the time particularly in those with low damage levels (Figure 1). Human AGS cells treated with amoxicillin, an oxidative stress inducer, were used in the experiment as positive control (lane A of Figure 1).

### 3.2. Individual Variation of Oxidative DNA Damage of Lymphocytes of Peripheral Blood

**Figure 2:**
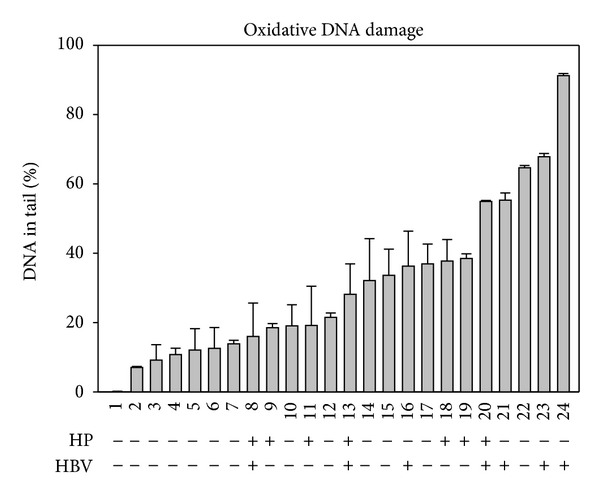
Variation of oxidative DNA damage levels of lymphocytes from 24 blood samples. Oxidative DNA damage levels of the lymphocytes isolated from 24 blood samples obtained from individuals who participated in health checkup. The oxidative DNA damage levels, represented as % DNA in tail, were aligned in the order from low to high, and each sample was coded (1–24) based on the respective DNA damage level. Carriers and noncarriers of HP or HBV are indicated with + or −, respectively.

The oxidative DNA damage levels of twenty-four blood samples randomly collected from individuals for ordinary health checkup were measured. The tested samples were given labels according to the respective oxidative DNA damage levels in the order from low to high (see Figure 2), showing that the variation can be up to 10 folds. 

The levels of comet assay without EndoIII/Fpg, determined for 10 of the 24 blood samples, were, as expected, very small (1.58 ± 1.3% DNA in tail). Such levels are negligible if compared with the levels determined by comet assay with EndoIII/Fpg. 

Among the tested samples, those from the individuals with infection of *Helicobacter pylori* (HP) or carriers of *hepatitis B virus* (HBV) were identified after the measurement of oxidative DNA damage levels. The samples from HP or HBV carriers apparently tend to be at the right-handed side, that is, the side with high oxidative DNA damage levels. This is supported by comparing the means of the oxidative DNA damage levels: 31.0 ± 5.0% for HP carriers; 51.1 ± 9.2% for HBV and 21.5 ± 4.6% for noncarriers (*P* < 0.05). Thus, lymphocytes from HP or HBV carriers have significantly higher oxidative DNA damage than those from noncarriers.

### 3.3. Individual Variation of Oxidative DNA Damage of Lymphocytes of Peripheral Blood Is Not Correlated with the Antioxidant Capacity of Serum

**Figure 3:**
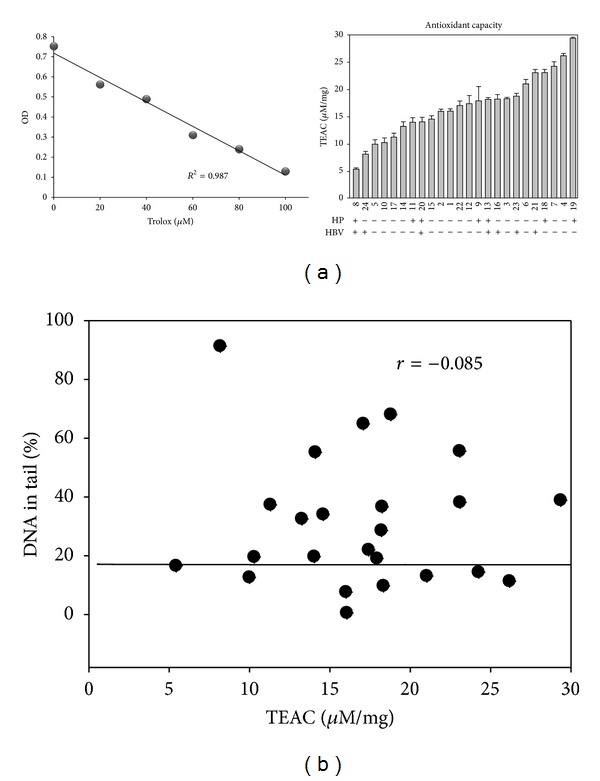
Variation of antioxidant capacities of sera from 24 blood samples. (a) Antioxidant capacities of sera isolated from 24 blood samples as described in Figure 2. Top part: trolox standard curve. Bottom part: The sample code was given according to those described in Figure 2, and the carriers and noncarriers of HP or HBV are indicated with + or −, respectively. (b) Correlation between the oxidative DNA damage levels described in Figure 2 and the antioxidant capacities of Figure 3(a). The *r* value (= −0.085), the correlation coefficient, was obtained from statistical analysis.

To explore the factors that influence the oxidative DNA damage level of lymphocyte, the antioxidant capacity of serum was measured and the respective measurements were also shown in the order from low to high, while the sample labels remain the same as those given previously (Figure 3(a)). Since the order of antioxidant capacity is no longer 1 to 24, it is readily to conclude that there is no correlation (either positive or negative) between oxidative DNA damage level and antioxidant capacity of serum. In addition, the variation of antioxidant capacities among serum samples is at most 6 folds, not as dramatic as that with oxidative DNA damage. This conclusion is also true if the oxidative DNA damage level is plotted against the antioxidant capacity shown in Figure 3(b). 

Moreover, the antioxidant capacities of serum samples from HP or HBV carriers do not show any preference to low or high side (Figure 3(a)), suggesting that antioxidant capacities of serum is not greatly affected due to the infection with either HP or HBV. 

### 3.4. *Helicobacter pylori* or *Hepatitis B* Carriers Have Lower NE Activity to Repair Oxidative DNA Damage

**Figure 4:**
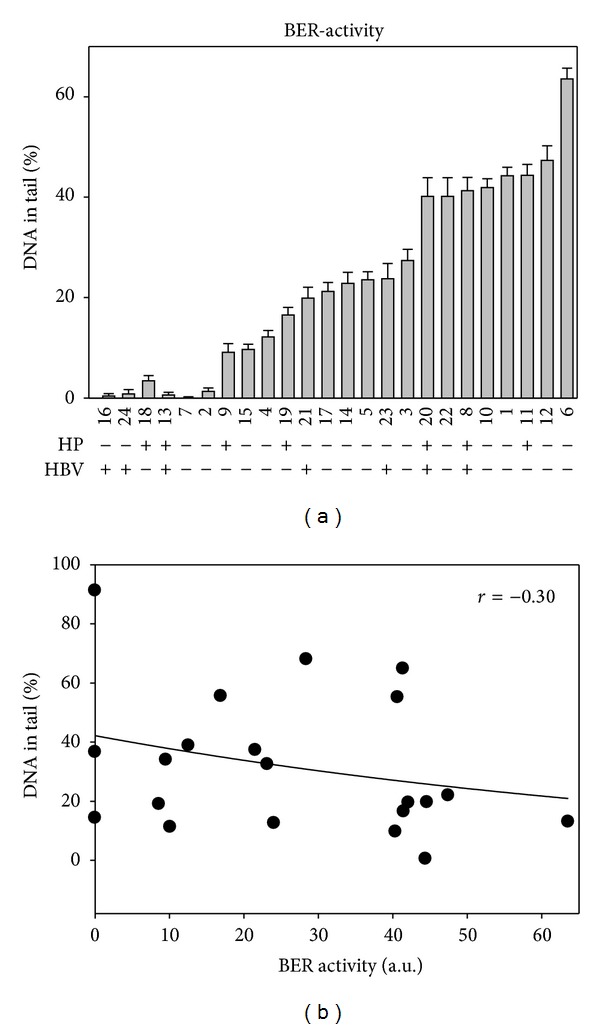
Variation of excision activities in nuclear extracts of lymphocytes from 24 blood samples. (a) To test the lymphocyte's nuclear etract's (NE's) activity, the AGS cells were served as substrate. AGS cells were treated with 5 mM amoxicillin to induce oxidative DNA damage and examined by comet-NE assay with lymphocyte's NE. The sample code was given according to those described in Figure 2, and the carriers and noncarriers of HP or HBV are indicated with + or −, respectively. (b) Correlation between the oxidative DNA damage levels of Figure 2 and the excision activities of Figure 4(a) (*note*: samples coded 2, 13 and 18 were excluded from the correlation because the concern of artifact due to sample preparation; their respective NER activities shown in Figure 5(a) are also low). The *r* value (= −0.30), the correlation coefficient, was obtained from statistical analysis.

To explore whether the repair capacity plays any role in the oxidative DNA damage level of lymphocyte, the activity to repair amoxicillin-induced DNA damage was assayed for the nuclear extracts of lymphocyte of tested blood samples. Amoxicillin has been shown by us to cause oxidative stress [8, 20]. The results of the repair activity assay shown in Figure 4(a) indicate that the variation among the tested samples can be more than 10 folds, as dramatic as the variation of oxidative DNA damage levels. The repair activity and the oxidative DNA damage level are weakly correlated in negative way (*r* = −0.30; see Figure 4(b)).

**Figure 5:**
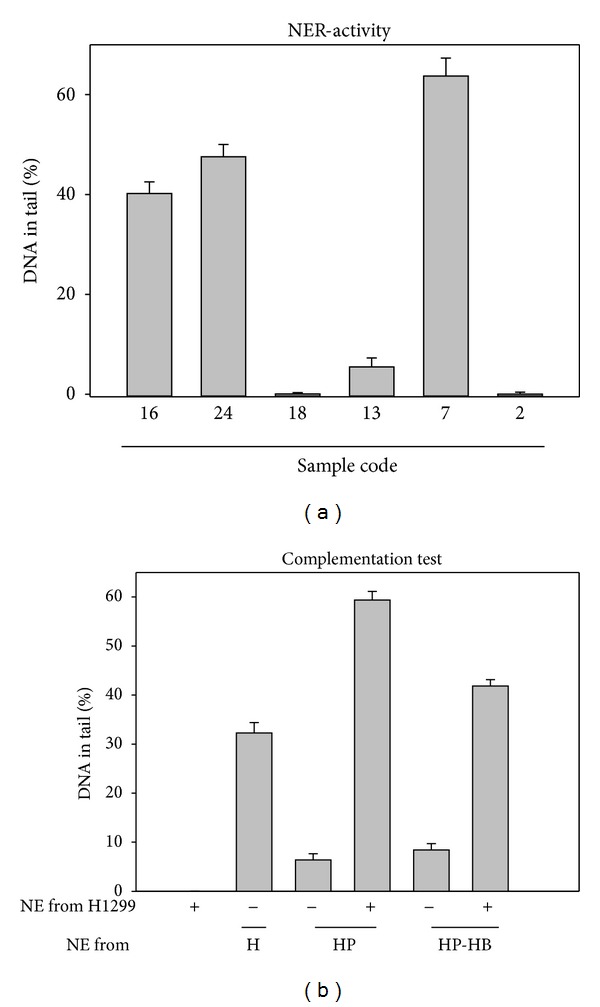
Control experiments for excision activities measured by the experiment described in Figure 4(a). (a) Nucleotide excision activities in some nuclear extracts of lymphocytes from the 24 blood sample. Nuclear extracts which failed to excise amoxicillin-induced DNA lesions were selected and used for examining their activities to excise UV-induced lesions. To determine the nucleotide excision activity, AGS cells treated with 5 J/m^2^ UV were used as substrates in comet-NE assay. (b) Complementation test. Nuclear extracts samples from *Helicobacter pylori* carrier (HP) and HP-HBV (HP-HB) double carrier were mixed with the NE from p53 deficient cell line H1299 and used as in comet-NE assay. The NE from H1299 cell and healthy (H) people were severed as negative and positive control.

To test the possibility that the low activity levels (Figure 4(a)) may be the artifact of nuclear extract preparation, the respective activities to repair UV-induced DNA damage were assayed. The results indicate that some of these NE samples (e.g., samples coded 16 and 7) exhibited high NER activities (Figure 5(a)), suggesting that their low BER activities cannot be attributed to the artifact during NE preparation. Also, complementation of the repair activity could be seen when the NE samples with undetectable activities were mixed (Figure 5(b)). 

Moreover, it is noted that repair activities in NE samples from HP or HBV carriers are lower than those of noncarrier: 18.9 ± 4.6% versus 29.38 ± 5.4% (mean ± SE; *P* < 0.05). 

## 4. Discussion

### 4.1. The Oxidative Damage Level of Human Lymphocyte Is Not Correlated with the Serum Antioxidant of Capacity or the Base Repair Excision Activity in Nuclear Extracts of Lymphocyte

To know if the oxidative damage level of human lymphocyte is correlated with the serum antioxidant of capacity, we first examined if the individual oxidative DNA damage level of lymphocyte is steady over a period of time, for example, weeks or months. Previous studies about the oxidative damage level of human lymphocyte mostly showed the values of single time point. Our study based on the results of four individuals indicates that the oxidative DNA damage levels of lymphocytes are steady at least for one month. The results suggest that the daily meals, particularly food taken occasionally or medicine administered probably, have little influence on the oxidative DNA damage level of lymphocyte. Since the damage level varied among the individuals, the genetic or pathological background of chronic illness appears to be decisive. On the other hand, the serum antioxidant capacity has been shown to be not affected by the sampling time [21]. Our results show that the level of oxidative DNA damage in PBL is not correlated with the serum antioxidant capacity but is weakly correlated with the BER activity in lymphocyte NE; in other words, DNA damage repair activity of lymphocyte but not serum antioxidant capacity may play some role in determining the steady state of oxidative DNA damage level of lymphocyte. 

The oxidative DNA damage levels in lymphocytes may be measured as the levels of 8-oxo-7,8-dihydro-2′-deoxyguanosine (8-oxodG) with HPLC or the EndoIII/Fpg sensitive sites with comet assay. Similar to our observation, marked variations in oxidative DNA damage levels among individuals have been noted by the previous investigators [22–25]. The levels of 8-oxodG among the healthy individuals can vary about 10 folds [24]. Previous study has indicated that the mean levels of oxidative DNA damage of PBL varied with different population [24], supporting that genetic background plays role in this regard. Other studies have indicated that diseases such as cancer, diabetics, and cardiopathologic condition increase the mean of levels oxidative DNA damage of PBL [26, 27]. Collins et al. [28] have shown that oxidative DNA damage levels are positively correlated with colorectal cancer in men and negatively correlated with stomach cancer in women.

The antioxidant capacity in our study is total antioxidant capacity (expressed in TEAC), which is contributed by various components of serum including proteins, lipids, and small molecules. Previous study has indicated that proteins and urate contribute about 52% and 38% of total antioxidant capacity, respectively [29]. Since serum concentrations of proteins and urate are usually not affected by short-term dietary treatment, the total antioxidant capacity would be little influenced by food or other nutrient supplements. Although it is more precise to know the antioxidant capacity of individual component in serum, it is technically more challenging to do so as separation and identification of the individual component are necessary. Actually, the correlation between plasma levels of antioxidants such as *α*-tocopherol or *β*-carotene or ascorbate and endogenous oxidative DNA damage has been found insignificant [30]. On the other hand, the overall or total antioxidant capacity (TEAC used in our study) is wildly used in previous studies. Previous study has shown that TEAC is comparable with other similar methods, indicating little variation (<10%) among the total antioxidant capacities of healthy individuals [29]. Thus, TEAC assay is a relevant assay for our purpose. 

The conclusion in the present study *should not* be interpreted that dietary supplement of antioxidant has no influence on the level of oxidative DNA damage in PBL. In fact, Duthie et al. [30] have reported that the oxidative DNA damage levels are not correlated with the plasma levels of *α*-tocopherol or *β*-carotene or ascorbate of individuals either smokers or nonsmokers of normal healthy males. This is consistent with our conclusion: no correlation between oxidative DNA damage levels and TEAC levels. However, in their study, supplement of diet for 20 weeks with vitamin C, vitamin E, and *β*-carotene reduce the endogenous oxidative DNA damage in PBL (from 60.5 ± 7.0 to 38.0 ± 5.9.). It should be noted that the reduction is less than 2 folds, suggesting that the dietary effect is not enough to explain the wild variation of endogenous oxidative DNA damage in PBL. Thus, our study does not go against the findings by Brevik et al. [31], who have reported that dietary supplement with some plant products increases BER by 55% and decreases NER by 39%, with kiwifruit NER (−38%) and BER (no effect). The two studies are of different context. 

### 4.2. The Oxidative Damage Levels of Lymphocytes and the Base Excision Repair Capacity from *Helicobacter pylori* (HP) or *Hepatitis B Virus* (HBV) Carriers

Our results suggest that individuals infected with HP or HBV tend to have higher oxidative DNA damage levels in their lymphocytes. Such observation is consistent with the conclusion of the previous reports that infection of HP increases the DNA damage of lymphocyte [32] and is in agreement with the clinic observation that carriers of HP or HBV have higher risk of cancer affliction [33, 34]. Our study suggests that the reduced DNA damage repair activities but not antioxidant capacities are more relevant to their high oxidative DNA damage levels. Why the infection of HP or HBV impairs the repair capacity of lymphocyte is unclear. The effect of HP infection on BER has been studied. APE1, the human endonuclease to process abasic sites formed during BER, may be downregulated or upregulated depending on the experimental conditions [35, 36]. As reactive oxygen species have been well known to be associated with HP infection, the role of BER to protect against tumorigenesis due to HP infection has been recognized. On the other hand, the recent study indicates that X protein of HBV, which structurally resembles human thymine DNA glycosylase (TDG), a key enzyme of BER, inhibits TDG-dependent BER [37]. Our preliminary study on repair activity complementation shows promising for the kind of research to explore the molecular mechanism leading to low BER activity. 

## 5. Conclusion

In this research, we have demonstrated that the lymphocyte's DNA oxidative level is steady more than one month. The level of oxidative DNA damage in PBL is not correlated with the serum antioxidant capacity but is weakly correlated with the BER activity in lymphocyte NE. Our study shows that lymphocytes of blood samples from carriers of *Helicobacter pylori or hepatitis B virus*, as compared to those of noncarriers, have higher oxidative DNA damage levels and lower repair activities.
